# Identification of Five Hub Genes as Key Prognostic Biomarkers in Liver Cancer via Integrated Bioinformatics Analysis

**DOI:** 10.3390/biology10100957

**Published:** 2021-09-24

**Authors:** Thong Ba Nguyen, Duy Ngoc Do, Tung Nguyen-Thanh, Vinay Bharadwaj Tatipamula, Ha Thi Nguyen

**Affiliations:** 1 Department of Anatomy, Biochemistry, and Physiology, John A. Burns School of Medicine, University of Hawaii at Manoa, Honolulu, HI 96813, USA; nguyenb7@hawaii.edu; 2 Department of Animal Science and Aquaculture, Dalhousie University, Truro, NS B2N 5E3, Canada; 3 Institute of Biomedicine, Hue University of Medicine and Pharmacy, Hue University, Hue 530000, Vietnam; nguyenthanhtung@hueuni.edu.vn; 4 Faculty of Basic Sciences, Hue University of Medicine and Pharmacy, Hue University, Hue 530000, Vietnam; 5 Institute of Research and Development, Duy Tan University, Danang 550000, Vietnam; vinaybharadwajtatipamula@duytan.edu.vn; 6 Faculty of Medicine, Duy Tan University, Danang 550000, Vietnam

**Keywords:** liver cancer, TOP2A, RRM2, NEK2, CDK1, CCNB1, hub genes, prognostic biomarker

## Abstract

**Simple Summary:**

Liver cancer is one of the most common cancers; however, the molecular mechanisms of liver tumorigenesis and progression are not completely understood. In the current study, we combined several bioinformatic approaches (differential gene expression analyses, weighted gene co-expression network analysis, pathway and gene-disease network enrichment) to identify potential hub genes and molecular pathways that contribute to liver cancer onset and development. The results revealed DNA topoisomerase II alpha (TOP2A), ribonucleotide reductase regulatory subunit M2 (RRM2), never in mitosis-related kinase 2 (NEK2), cyclin-dependent kinase 1 (CDK1), and cyclin B1 (CCNB1) as the hub genes for liver cancer. Subsequent validation suggested TOP2A, RRM2, NEK2, CDK1, and CCNB1 as the prognostic biomarkers of liver cancer.

**Abstract:**

Liver cancer is one of the most common cancers and the top leading cause of cancer death globally. However, the molecular mechanisms of liver tumorigenesis and progression remain unclear. In the current study, we investigated the hub genes and the potential molecular pathways through which these genes contribute to liver cancer onset and development. The weighted gene co-expression network analysis (WCGNA) was performed on the main data attained from the GEO (Gene Expression Omnibus) database. The Cancer Genome Atlas (TCGA) dataset was used to evaluate the association between prognosis and these hub genes. The expression of genes from the black module was found to be significantly related to liver cancer. Based on the results of protein–protein interaction, gene co-expression network, and survival analyses, DNA topoisomerase II alpha (*TOP2A*), ribonucleotide reductase regulatory subunit M2 (*RRM2*), never in mitosis-related kinase 2 (*NEK2*), cyclin-dependent kinase 1 (*CDK1*), and cyclin B1 (*CCNB1*) were identified as the hub genes. Gene Ontology and Kyoto Encyclopedia of Genes and Genomes pathway enrichment analyses showed that the differentially expressed genes (DEGs) were enriched in the immune-associated pathways. These hub genes were further screened and validated using statistical and functional analyses. Additionally, the TOP2A, RRM2, NEK2, CDK1, and CCNB1 proteins were overexpressed in tumor liver tissues as compared to normal liver tissues according to the Human Protein Atlas database and previous studies. Our results suggest the potential use of *TOP2A, RRM2,* *NEK2, CDK1,* and *CCNB1* as prognostic biomarkers in liver cancer.

## 1. Introduction

Liver cancer is the sixth most common cancer and the fourth leading cause of cancer mortality, with 2.09 million new cases and 1.76 million deaths recorded globally in 2018 [1]. Hepatocellular carcinoma (HCC), a major form of primary liver cancer, accounts for ~80% of all primary liver cancer cases [1,2]. Due to lack of specific clinical appearances in the early stages, most of the patients with primary liver cancer are diagnosed at advanced stages with fewer treatment options, resulting in poor prognosis and outcomes [3]. Despite the recent advances in cancer biology and genetic profiling, the molecular pathogenesis of HCC is still not fully understood. Therefore, a deep understanding of cancer pathogenesis may aid in early diagnosis and treatment, thereby improving the overall survival (OS) of patients with liver cancer. The identification of the key genes and/or biological pathways regulating tumor proliferation and progression using different bioinformatics tools is crucial to discover the molecular mechanisms underlying cancer development. Consequently, this knowledge can be used to develop new biomarkers or treatment methods to improve the outcomes of patients with liver cancer. Gene expression profiling of cancer can serve as an independent survival predictor and contributes to the treatment options [4,5,6,7,8].

Weighted gene co-expression network analysis (WGCNA) is a common bioinformatics approach for the identification of modules of highly inter-correlated genes. This method is largely used in numerous biological processes, typically for the detection of candidate diagnostic and/or therapeutic targets for different malignant tumors [9]. In the current study, a co-expression network was built via WGCNA to identify the morphology-specific modulators of liver cancer based on the transcriptional profile of a liver cancer dataset GSE14520 extracted from the Gene Expression Omnibus (GEO) database [10]. Gene set enrichment (GSE) analysis was conducted to find the potential functions of these hub genes. Moreover, these hub genes were screened out by univariate Cox regression analysis and assessed for correlation with methylation status, thus providing highly accurate analytic results. The Cancer Genome Atlas (TCGA) database was then used to identify the potential prognostic biomarkers of liver cancer [11]. Subsequently, the protein levels of the identified genes were checked using the Human Protein Atlas (HPA) database and previous studies to see if they are upregulated in tumor tissues. This knowledge provides new insights into the potential molecular mechanisms of liver cancer.

## 2. Materials and Methods

### 2.1. Dataset Collection

**Figure 1:**
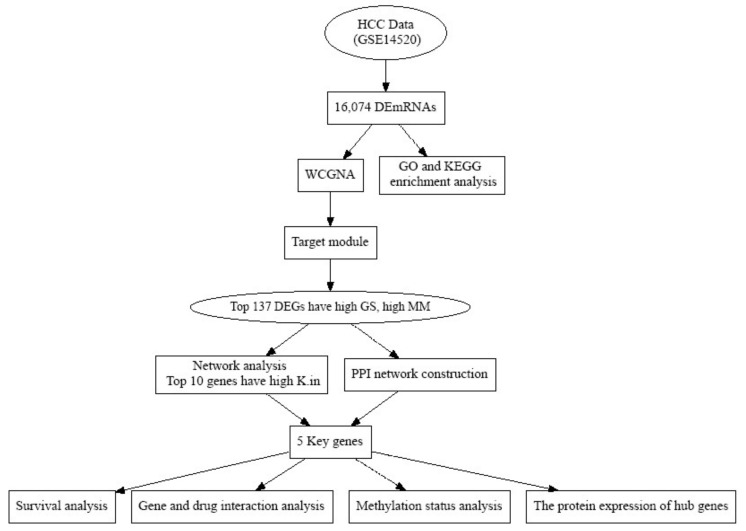
The flow chart of data collection, processing, analysis, and validation. HCC: hepatocellular carcinoma; DEmRNAs: differentially expressed mRNAs; PPI: protein–protein interaction; GSE: gene expression data; GO: gene ontology; KEGG: Kyoto Encyclopedia of Genes and Genomes; WGCNA: weight gene co-expression network analysis; MM: module membership; GS: gene significant; K.in: intramodular connectivity.

The workflow of the current work is shown in Figure 1. Gene expression profiles of dataset GSE14520 were obtained from the GEO database (https://www.ncbi.nlm.nih.gov/geo/query/acc.cgi?acc=GSE14520 (accessed on 15 January 2021)). This dataset comprises the mRNA expression data of 220 normal tissue samples and 225 HCC samples (Figure 1). Additionally, a total of 347 HCC and 50 normal liver tissue samples with detailed clinical information were obtained from the TCGA database as previously described [11].

### 2.2. Datasets Preprocessing and Differential Gene Expression Analysis

Prior to the differential expression analyses, a matrix of gene expression values was transformed using log2 function in R program, and then the values were presented as log2 transformed values (Table S1) [12]. Then, a principal component analysis was performed using prcomp function to check for potential outliers from the gene expression matrix. To ensure the quality of the data, only genes (probes) that were expressed in at least three samples were included for further analyses [12]. Differential expression analyses were performed using the Limma package [13]. The empirical Bayes procedure in the package was used to compare the expression level of genes between HCC and normal tissues [14]. For statistical analyses, *p*-values were adjusted using the false discovery rate (FDR) correction method, and only genes with adjusted *p*-values < 0.05 were denoted as DEGs.

### 2.3. Weighted Gene Co-Expression Network Construction

To reduce computational requirements and to keep the meaningful genes in the network construction, only the DEGs were used as the input for WGCNA analyses. The WGCNA methodology was adapted from a previous study [15]. Briefly, an adjacency matrix was created (using the Pearson’s correlations between all genes) and raised to a power β of 9. The module membership (MM) was calculated by using the WGCNA function signedKME; where deep split = 2, minModuleSize = 30. A hierarchical clustering tree was constructed based on the correlation matrix, dissimilarity metrics, and the gene co-expression of different nodes in order to organize samples into desired clusters. The dynamic tree cutting method was applied to pinpoint more precisely the significant co-expression modules [16]. After that, a target module that was highly correlated with a particular phenotype or condition/disease can be extracted from the tree. The hub genes, which showed a higher value of internal connectivity and a significant association between genes and feature vector in the target module, were then identified [15,16].

### 2.4. Module–Trait Relationship Analysis of Liver Cancer

The correlation between HCC and modules were assessed by Pearson’s correlation tests by attributing normal people and cancer patients to a value of 0 and 1, respectively. The module eigengene (ME) represents the common expression value of all of the genes of each module. The MM value is the association of ME and the gene expression profile (MMi = │cor(x(i)), ME│; where i is the value of each gene). The closer the MM value of a gene to 1, the more important that gene is in a given gene module. Gene significance (GS) value represents the correlation between HCC and the genes (GS = │−log(p)│; where *p* is the value of the Student’s *t*-test). The intramodular connectivity (K.in) value was the average connection value of all of the genes within a module [16]. Detection of hub genes was usually based on the values of three main factors: the GS, MM, and K.in. The DEGs with GS and MM values larger than 0.2 and 0.8, respectively, were first selected as the potential hub genes [17,18]. These genes were then sorted based on their K.in value, and the ten genes with the highest K.in value were selected for gene regulatory network analysis. As a result, this method helped to reduce the dimensional issues, thereby improving cancer prediction and novel biological significance.

### 2.5. Function Enrichment Analysis

Gene Ontology (GO) and Kyoto Encyclopedia of Genes and Genomes (KEGG) pathway enrichment analyses were performed for all DEGs using the clusterProfiler package [19]. GO terms include three factors: biological process, cellular component, and molecular function. While GO was used to explore the function of genes in biological systems, KEGG was used to identify the signaling pathways of DEGs [20]. A *p*-value of 0.05 was utilized as a cut-off.

### 2.6. Gene Regulatory Network

A gene regulatory network could be used to evaluate the interaction between genes within the network in order to identify the potential genes of unknown signaling pathways. Network analysis of the top genes in the significant module was done using the R package igraph and qgraph [21,22]. Nodal strength is calculated as the sum of the edge weights within a network. Higher values of nodal strength demonstrate a faster and more direct effect on other nodes. The node strength centrality in the networks is essential to identify functionally important genes [23,24,25]. Network analysis was performed using extended Bayesian information criteria selection [26] and the glasso algorithm [27]. Genes with the highest node strength centralities were identified as the key genes [28].

### 2.7. Protein–Protein Interaction Network Construction

The DEGs with GS > 0.2 and MM > 0.8 in the best module were used to build a protein–protein interaction (PPI) network using Search Tool for the Retrieval of Interacting Genes (STRING) and were visualized through CYTOSCAPE software (http://www.cytoscape.org; latest version 3.8.2; accessed on 20 August 2021). MCODE score > 2, number of nodes > 3, and medium confident interaction score > 0.4 were set as cut-off criteria for module identification and network visualization. Degree > 67 was selected as the cut-off criterion for the key genes.

### 2.8. Methylation Analysis

The gene expression and methylation of five hub genes in HCC were evaluated using the UALCAN tool. It is a user-friendly web resource for analyzing cancer data and providing information on DNA methylation and gene expression levels [29].

### 2.9. Survival Analysis

The data of 347 patients with HCC obtained from TCGA was accessed. Based on the median value of the prognostic risk score, these HCC patients were allocated into low-risk and high-risk groups to perform survival analysis. Kaplan–Meier curves were drawn, and the correlations between the DEGs and OS were evaluated by univariate Cox regression analysis. The hazard ratio (HR) of death and adjusted *p*-values were computed by using Bonferroni correction of Cox proportional hazards analysis [30]. An adjusted *p*-value < 0.05 was considered statistically significant. Additionally, survival analysis of the hub genes was also performed by using OSlihc, an online tool, as previously described [31].

### 2.10. The Protein Expressions of the Prognostic Hub Genes

To assess the translational levels of the five hub genes, we attained immunohistochemistry (IHC) sections of normal liver tissue and HCC tissue samples from the Human Protein Atlas database (HPA) [32] and two previous studies [2,33].

### 2.11. Gene–Drug Interaction Analysis

The possible interaction of the currently available drugs with five hub genes was explored through the drug–gene interaction database (DGIdb) and visualized through CYTOSCAPE software (http://www.cytoscape.org (accessed on 20 March 2021); latest version 3.8.2).

## 3. Results

### 3.1. Key Modules Identification by Weighted Gene Co-Expression Network

After preprocessing the data, the expression matrices of 22,268 genes were obtained from 445 samples. By using a cutoff of *FDR* < 0.05, a set of 16,074 DEGs was identified (Figure 1). The DEGs between liver cancer and normal control samples from TCGA data are presented in Figure S1. The power of β = 9 was designated as the soft-threshold factor to perform a scale-free network (Figure S2). Twenty-six co-expression modules comprising from 33 to 7105 DEGs were identified (Table 1) and represented as 26 different unique colors (Figure S2). A larger correlation and smaller *p*-value indicated a stronger association between the module and HCC. Accordingly, the most interesting modules were the black module (*r* = 0.872, *p* < 0.001) and the light-green module (*r* = −0.711, *p* < 0.001). The black module presented the largest correlation that met a cutoff of 0.8 and *p* < 0.001; it was speculated to play important roles in the pathophysiology of HCC and was subjected to successive analyses (Table 1).

### 3.2. Identification of Hub Genes through Gene Regulatory Networks

The black module comprises 656 genes (Table 1). Notably, a hub gene usually has a high GS, high MM, and high K.in. By overlapping the genes of the black module with identified DEGs and applying the cutoff of GS > 0.2 and MM > 0.8, the top 137 genes were identified. Afterward, the top ten genes with the highest K.in value were selected, namely ribonucleotide reductase regulatory subunit M2 (*RRM2*), DNA topoisomerase II alpha (*TOP2A*), replication factor C subunit 4 (*RFC4*), never in mitosis-related kinase 2 (*NEK2*), H2A histone family member X (*H2AFX*), DNA primase polypeptide 1 (*PRIM1*), dumbbell former 4 protein (*DBF4*), centromere protein (*CENPA*), kinesin family member 14 (*KIF14*), and FA complementation group I (*KIAA1794*) (Table 2).

**Figure 2:**
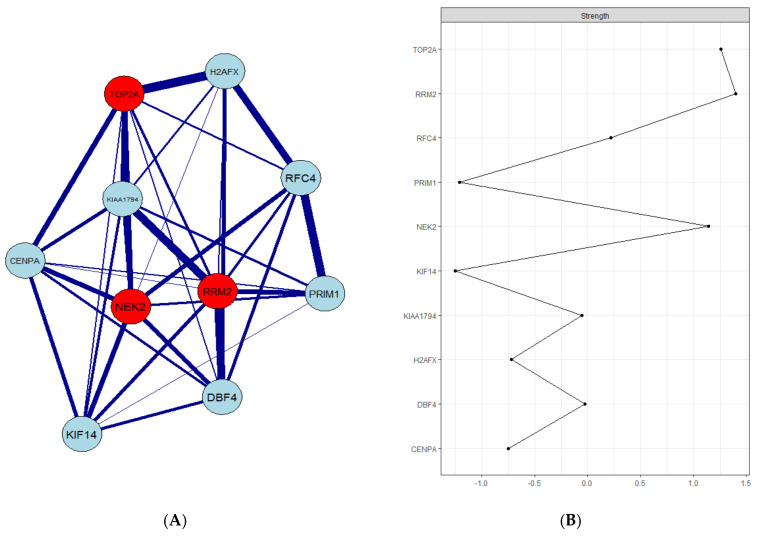
Co−expression network of the top ten genes of the black module: (**A**) each node in the co−expression network denotes a gene; nodes with red signify the hub genes, and the middle line represents the link between genes. (**B**) The connection strength of the top ten genes.

The relationship between target genes and other hub genes of the module was presented in a gene co-expression network. Among those, three hub genes denoted in red, namely *TOP2A*, *RRM2*, and *NEK2,* have the highest degree scores in the network (Figure 2).

### 3.3. PPI Network Construction and Hub Gene Validation

We explored the PPI interactions network by STRING database of the proteins encoded by the top 137 DEGs in the black module (Figure S3). The PPI network topological analysis revealed three top proteins, namely CDK1 (cyclin-dependent kinase 1), CCNB1 (cyclin B1), and TOP2A, that were noted to meet the cut-off criterion of degree > 67 (Table S2). Of these, only *TOP2A* was in the list of top 10 genes with the highest K.in values. Four modules for potential hub genes in the PPI network satisfied the MCODE score > 2 and the number of nodes > 3 cut-offs (Figure S4).

### 3.4. Functional and Pathway Enrichment Analysis

**Figure 3:**
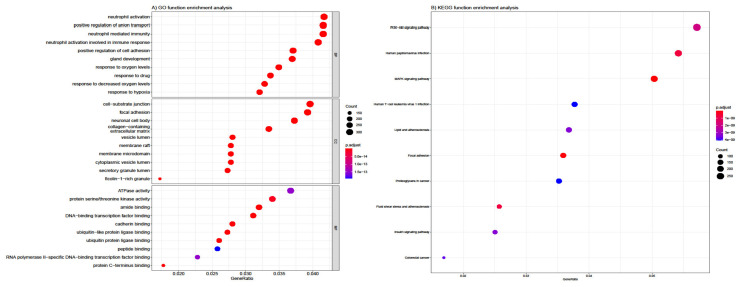
GO (**A**) and KEGG (**B**) function enrichment analysis. The abscissa signifies the number of genes enriched in the Figure.

The top enriched biological process from GO included neutrophil activation, neutrophil-mediated immunity, neutrophil activation involved immune response, and neutrophil degranulation (Figure 3A). For cellular components, DEGs were chiefly associated with the neuronal cell body, cell-substrate junction, focal adhesion, and collagen-containing extracellular matrix. Lastly, for molecular function, DEGs were mostly involved in cell adhesion, DNA-binding, transcription factor binding, protein serine/threonine kinase activity, etc. A heatmap showed significant ontological processes between DEGs and GO terms (Figure 3A). KEGG analysis showed that DEGs were primarily enriched in the signaling pathways of PI3K (phosphatidylinositol 3-kinase)/AKT (protein kinase B), mitogen-activated protein kinase (MAPK), human T-cell leukemia virus 1 and human papillomavirus infection (Figure 3B).

### 3.5. Real Hub Genes Identification and Validation

**Figure 4:**
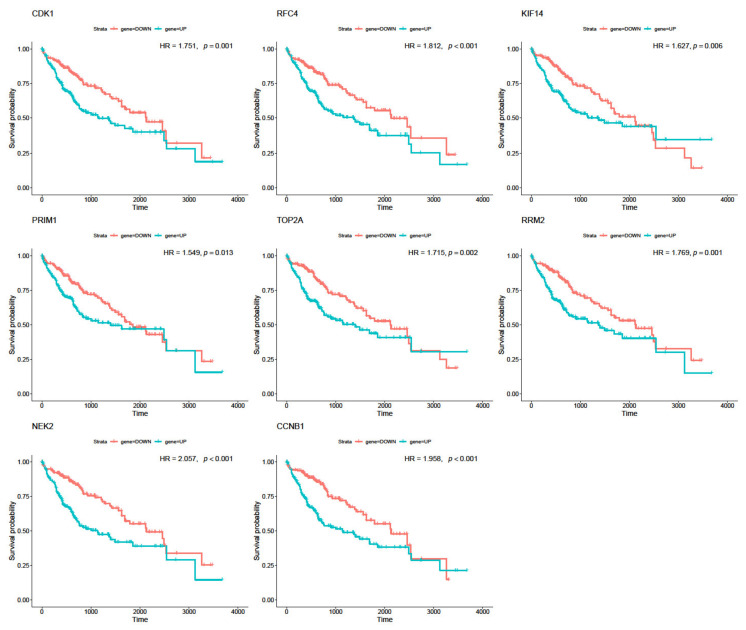
Survival analysis of five potential hub genes obtained from gene co-expression and PPI networks and three other genes of the top ten genes with the highest intramodular connectivity (*RFC4*, *KIF14*, and *PRIM1*) in HCC. Overall survival of the hub genes in HCC is based on Kaplan–Meier plotter. The horizontal axis represents the time to event (in days). The patients were allocated into the high-risk and low-risk groups and assigned a color. The red line designates the samples with low risk, and the green line represents the samples with high risk. *p* < 0.05 indicates a statistically significant difference in mortality between groups. HR: hazard ratio of the two groups.

The survival analysis using univariate Cox analysis was performed for five potential hub genes (*TOP2A*, *RRM2*, *NEK2, CDK1*, and *CCNB1*) obtained from gene co-expression and PPI networks, and three other genes of the top ten genes with the highest intramodular connectivity (*RFC4*, *PRIM1*, and *KIF14*) in HCC. The results exposed the significance of the five potential hub genes as prognostic factors for patients with liver cancer (Figure 4). Specifically, the high expression levels of *TOP2*A (*p* = 0.002), *RRM2* (*p* = 0.001), *NEK2* (*p* < 0.001), *CDK1* (*p* = 0.002), and *CNNB1* (*p* < 0.001) were identified as being strongly associated with poorer prognosis. Moreover, liver cancer patients with an increased expression level of *KIF14* (*p* = 0.006), *PRIM1* (*p* = 0.013), and *RFC4* (*p* < 0.001) also had poorer outcomes (Figure 4). The HR of death of the two groups ranged from 1.549 to 2.057 for all eight of the tested genes and from 1.715 to 2.057 for five potential hub genes, indicating a strong association between the expression of hub genes and the HR of death (Figure 4). In other words, patients with higher expression levels of *TOP2A*, *RRM2*, *NEK2, CDK1*, and *CCNB1* have significantly shorter survival periods than the patients with lower expression levels of these genes (Figure 4; Table S3). Moreover, the additional survival analysis using Oslihc further confirmed the significance of these hub genes in the OS of HCC patients (Figure S5).

### 3.6. The Protein Expression of Hub Genes

**Figure 5:**
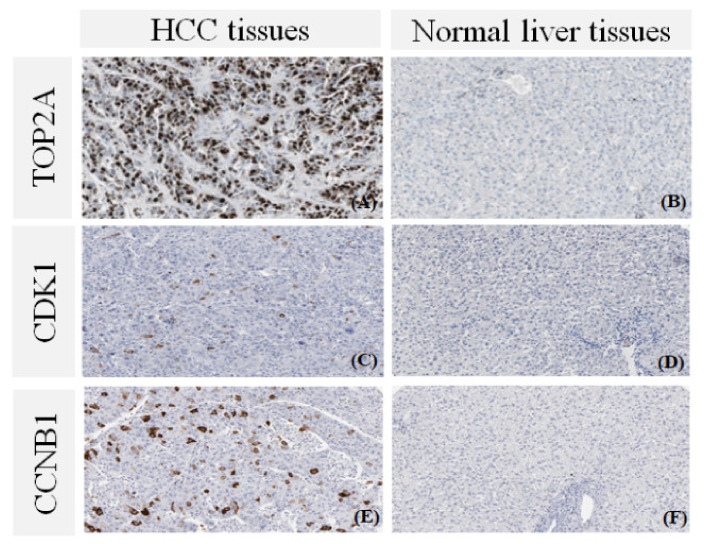
Immunohistochemistry of the five potential hub genes in liver cancer (HCC) and normal tissues from the Human Protein Atlas (HPA) database and previous studies [2,33]. Protein levels of (**A**) TOP2A in HCC tissue; (**B**) TOP2A in normal liver tissue; (**C**) CDK1 in HCC tissue; (**D**) CDK1 in normal liver tissue; (**E**) CCNB1 in HCC tissue; (**F**) CCNB1 in normal liver tissue.

The five candidate hub genes (*TOP2A, RRM2, NEK2, CDK1*, and *CCNB1*) were further investigated for their protein expression levels in HCC and normal liver tissues via the HPA database and previous studies. Accordingly, TOP2A, CCNB1, CDK1, RRM2 [2], and NEK2 [33] protein expression levels were substantially increased in HCC tissues samples as compared to that of normal liver tissues (Figure 5). Taken together, our results strongly indicated that liver cancer patients with an upregulated level of TOP2A, RRM2, NEK2, CDK1, and CCNB1 were associated with poor prognosis.

### 3.7. Hub Genes Expression Is Correlated with Methylation

**Figure 6:**
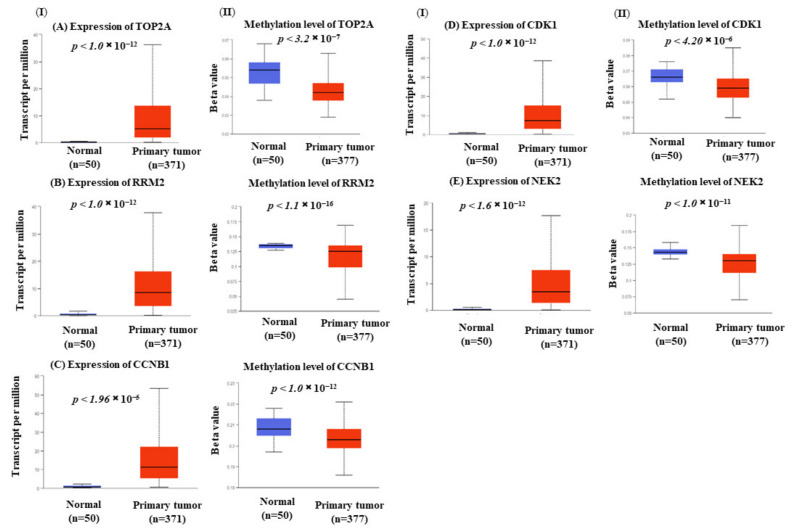
The status of the genes expression (I) and methylation (II) of the hub genes in liver cancer. The expression and promoter methylation pattern of (**A**) *TOP2A*, (**B**) *RRM2,* (**C**) *CCNB1,* (**D**) *CDK1,* and (**E**) *NEK2* in the primary liver tumors (*n* = 377) as compared to the normal samples (*n* = 50) using TCGA samples.

The gene expression and methylation expression patterns of five hub genes were assessed. Significant differences were observed in both the gene expression (Figure 6I) and methylation (Figure 6II) patterns of *TOP2A, RRM2*, *CCNB1, CDK1,* and *NEK2* when comparing liver tumor and normal liver tissues samples. Moreover, a negative association between gene expression and methylation patterns was also noted for all of these genes. This finding suggested that increased expression of the hub genes *TOP2A, RRM2, NEK2, CDK1*, and *CCNB1* in HCC might be a result of decreased DNA methylation levels in their encoded genes.

### 3.8. Gene–Drug Interaction Networks

Through the DGIdb, a total of 191 drugs related to five genes were selected. These drugs were found to be mostly related to the three genes *TOP2A, CDK1*, and *RRM2* (Figure S6).

## 4. Discussion

In the current study, we utilized WGCNA to identify novel biomarkers from 16,047 genes obtained from 445 samples of two datasets. We found 26 gene modules, with the number of eigengenes largely varying from 33 to 7105 DEGs. The striking correlations between genes in the module and clinical features may help to improve the current understanding of the pathogenesis of HCC. The black module appeared to comprise significant genes. The GO and KEGG pathway analyses revealed that the biological functions of the black module were strongly enriched for immune response. Enrichment function analysis demonstrated a contributory role of the inflammatory response in the development of HCC. The DEGs involved in neutrophil activation and neutrophil-mediated immunity were observed in both GO and KEGG analyses. Noticeably, the PI3K/AKT signaling pathway is commonly found to be hyper-activated in HCC, and inhibiting this pathway is one of the critical therapeutic approaches to treating HCC [34].

The combination of WGCNA, integrated bioinformatics, and PPI network identified *TOP2A, RRM2, NEK2, CDK1*, and *CCNB1* as the hub genes. Notably, *TOP2A* was found to be a top significant gene by WGCNA, network analysis, PPI network analysis, survival analysis, and IHC staining. *TOP2A* encoded for DNA topoisomerase II protein, which controls DNA topology during DNA replication [35]. Recently, the *TOP2A* gene was reported as a hub gene in HCC [36] with an inference value of 143.13 from the gene–disease association dataset in the Comparative Toxicogenomics Database, and it is currently being considered as a potential drug target for the treatment of HCC [37,38,39]. TOP2A has been shown to directly interact with P53, a well-known tumor suppressor protein [40]. Similarly, *RRM2* and *NEK2* were also noted as the highest-ranking genes by WGCNA and network analysis in this study. *RRM2* was well-known as a functional catalytic site in regulating cell cycle by controlling DNA repair and replication [41,42]. Alteration in RRM2 protein expression leads to the development of HCC [42]. *NEK2*, on the other hand, plays an important role in regulating mitotic processes [43]. *NEK2* has been highlighted as an oncogenic gene in various types of human cancers and is considered to be a potential therapeutic approach for human cancer treatment [44]. Moreover, NEK2 protein was shown to be important in FOXM1-related pathways that involve the dysregulation of HCC cell growth and apoptosis [45]. CDK1 is a central molecular regulator that control cells mitosis. Loss of CDK1 expression leads to the activation of Ras and the silencing of P53, thereby conferring resistance against tumorigenesis in liver cancer [46]. CCNB1, on the other hand, is a regulatory protein involved in cell proliferation. CCNB1 also interacts with the P53 signaling pathway and the cell cycle, which have been noted to be related to HCC [47]. Nevertheless, the biological interpretation of liver cancer using these potential prognostic biomarkers needs to be done with caution, as the results of enrichment analyses might suffer from potential bias caused by the proliferation genes in the background gene set [48].

According to the IHC staining from the HPA database and previous studies, TOP2A, RRM2, NEK2, CDK1, and CCNB1 protein expression levels were shown to be significantly increased in HCC tissues as compared to the normal liver tissues. DNA methylation is an important early event in tumor growth and progression [49]. In this study, we found the significantly increased expression of five hub genes and a negative correlation between the expression of these hub genes and their methylation status. This finding was in accordance with a previous study that showed an upregulation of *TOP2A* and *RRM2* and lower promoter methylation of these genes in cholangiocarcinoma [50].

Taken together, our results suggest *TOP2A, RRM2, NEK2, CDK1*, and *CCNB1* as HCC-associated hub genes that can serve as potential prognostic biomarkers in liver cancer. However, the hub genes identification of this study was mostly based on microarray gene expression data, which may require additional in vitro and in vivo functional tests before further confirmation and to shed light on their underlying molecular mechanisms.

## 5. Conclusions

In summary, we built a WCGNA, PPI network, gene regulatory network in order to detect and validate target genes as prognostic biomarkers for HCC. GO and pathway enrichment analyses indicated that the biological functions of the black modules were oriented toward immunity response. Moreover, our results demonstrated a significantly increased expression of *TOP2A, RRM2, NEK2, CDK1*, and *CCNB1* and the negative correlation between the expression of these genes and their methylation status in HCC. Finally, five hub genes, *TOP2A, RRM2, NEK2, CDK1,* and *CCNB1*, were noted as being significant genes. Further experimental studies are required to confirm their role as prognostic markers in HCC and to identify their molecular mechanisms of action in this type of cancer.

## Figures and Tables

**Table 1:** Correlation and *p*-value between each module and liver cancers after weight gene co-expression network analysis.

Module	Correlation	*p*-Value	Number of Genes
Black	0.872	<0.001	656
Blue	−0.104	0.028	1677
Brown	−0.663	<0.001	2396
Cyan	0.57	<0.001	220
Dark green	−0.042	0.382	65
Dark grey	0.677	<0.001	7105
Dark orange	0.503	<0.001	58
Dark red	0.506	<0.001	74
Dark turquoise	0.303	<0.001	59
Green yellow	0.4	<0.001	684
Grey	0.114	0.016	290
Grey60	−0.305	<0.001	487
Light cyan	−0.031	0.515	176
Light green	−0.711	<0.001	153
Light yellow	−0.374	<0.001	143
Magenta	−0.332	<0.001	455
Midnight blue	−0.112	0.018	189
Orange	−0.406	<0.001	58
Pale turquoise	−0.463	<0.001	33
Pink	−0.169	<0.001	563
Royal blue	0.393	<0.001	119
Saddle brown	−0.485	<0.001	40
Salmon	−0.017	0.713	232
Sky blue	0.407	<0.001	53
Steel blue	−0.447	<0.001	35
White	−0.109	0.021	54

**Table 2:** The top ten genes with the highest intramodular connectivity.

Genes	FC	Ave. Expr.	*t*	*p*-Value	Adj. *p*-Value	MM. Black	GS	Kin
*CENPA*	1.313	2.053	−20.644	4.89 × 10^−67^	2.46 × 10^−65^	0.881	0.699	182.908
*DBF4*	1.293	2.248	−23.814	1.30 × 10^−81^	1.61 × 10^−79^	0.883	0.748	188.790
*H2AFX*	1.291	2.683	−26.595	3.18 × 10^−94^	8.84 × 10^−92^	0.868	0.783	183.809
*KIAA1794*	1.294	2.135	−21.209	1.22 × 10^−69^	7.04 × 10^−68^	0.891	0.709	181.842
*KIF14*	1.249	2.063	−20.100	1.57 × 10^−67^	6.81 × 10^−63^	0.852	0.689	185.458
*NEK2*	1.468	2.136	−26.636	2.09 × 10^−94^	5.96 × 10^−92^	0.926	0.783	184.615
*PRIM1*	1.341	2.307	−24.049	1.10 × 10^−82^	1.47 × 10^−80^	0.853	0.751	186.033
*RFC4*	1.443	2.535	−29.438	8.27 × 10^−107^	5.17 × 10^−104^	0.910	0.812	181.994
*RRM2*	1.785	2.360	−31.691	1.62 × 10^−116^	1.81 × 10^−103^	0.929	0.832	184.521
*TOP2A*	1.686	2.292	−31.427	2.15 × 10^−115^	2.04 × 10^−112^	0.932	0.824	185.060

*RRM2:* ribonucleotide reductase subunit M2; *TOP2A:* topoisomerase II alpha; *RFC4:* replication factor C subunit 4; *NEK2:* never in mitosis-related kinase 2; *H2AFX:* H2A histone family member X; *PRIM1:* DNA primase polypeptide 1; *DBF4:* dumbbell former 4 protein; *CENPA:* centromere protein A; *KIF14:* kinesin family member 14; *KIAA1794:* FA complementation group I; FC: fold change; Ave. Expr.: average of gene expression; adj. *p*−value: adjusted *p*−values; *t*: *t* value of *t*−test; MM: module membership; GS: gene significant.

## Data Availability

All relevant data and codes that support the findings of this study are openly available on the Open Science Framework at http://doi.org/10.17605/OSF.IO/RNGDB (accessed on 6 September 2021).

## Supplementary Materials

The following are available online at https://www.mdpi.com/article/10.3390/biology10100957/s1: Figure S1: Volcano map of genes differentially expressed between liver cancer and normal samples from TCGA data; Figure S2: Determination of soft-thresholding power in weighted gene co-expression network analysis (WGCNA) analysis; Figure S3: Protein–protein interaction (PPI) network of hub genes that were identified by Cytoscape; Figure S4: Modules of protein–protein interaction (PPI) network; Figure S5: Survival analysis of five hub genes and three other genes of the top ten genes with the highest intramodular connectivity in HCC in OSlihc; Figure S6: Drug–genes interaction network; Table S1: Comparison of the log2(count) values of the top ten differentially expressed genes in HCC patients versus control; Table S2: Degree of proteins in the protein–protein network by Cytoscape; Table S3: The association of the hub genes and overall survival in the TCGA_LIHC dataset.
