# Supplementary material for: Identification of Five Hub Genes as Key Prognostic Biomarkers in Liver Cancer via Integrated Bioinformatics Analysis
Source: Biology (Basel). 2021 Sep 24;10(10):957. doi: 10.3390/biology10100957 (PMC8533228; doi:10.3390/biology10100957)
Supplement: Supplementary file 1 [file biology-10-00957-s001.zip › biology-1323233-supplementary.pdf]

Article

Identification of five hub genes as key prognostic biomarkers in liver cancer via integrated bioinformatics analysis

Supporting figures:

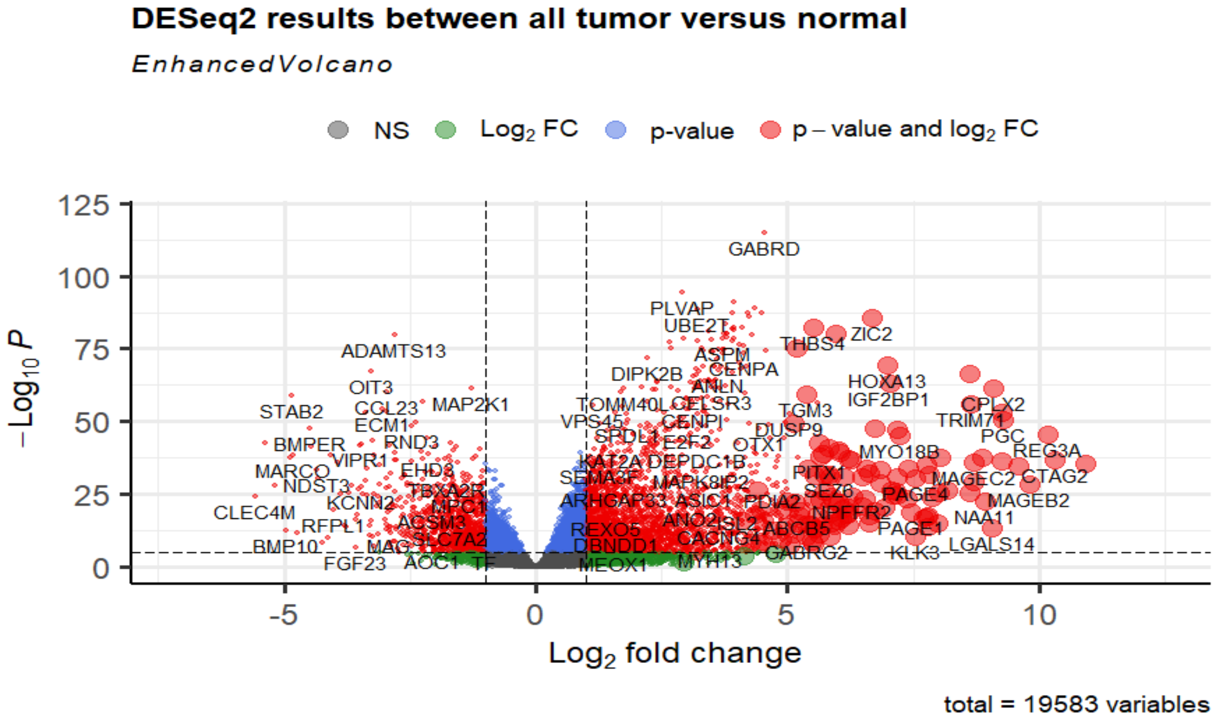

Figure S1: Volcano map of genes differentially expressed between liver cancer and normal samples from TCGA data. The x-axis represents the corrected p-value (scale conversion using logarithm), and the y-axis represents the fold change (log2FC). Each dot in the figure represents a gene; red or green dots represent genes that are significantly upregulated or downregulated, respectively, and black dots represent genes that have no differences in expression between cancer samples and healthy controls.

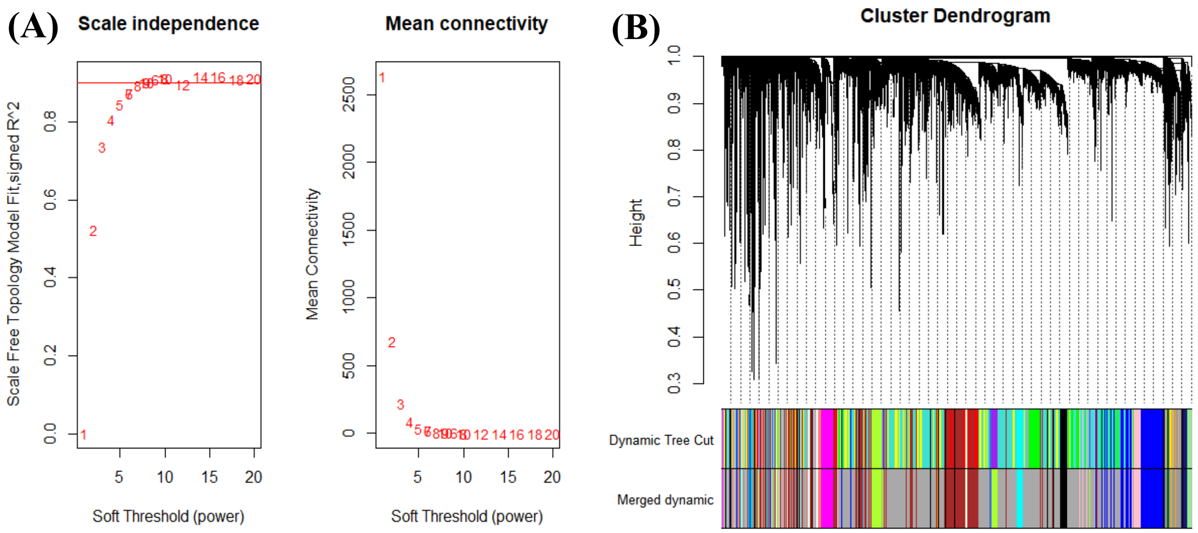

Figure S2: Determination of soft-thresholding power in weighted gene co-expression network analysis (WGCNA) analysis; (**A**) The scale-free fit index for various soft-thresholding powers β and the mean connectivity for various soft-thresholding powers. (**B**) A gene clustering tree (cluster dendrogram) produced by hierarchical clustering of adjacency-based dissimilarity. The colored row below the dendrogram indicates module membership identified by the dynamic tree cut method. The height represents similarity, and the correlation between height and similarity is negative. Each color represents a module, and each vertical line represents a gene. Twenty-six modules were identified.

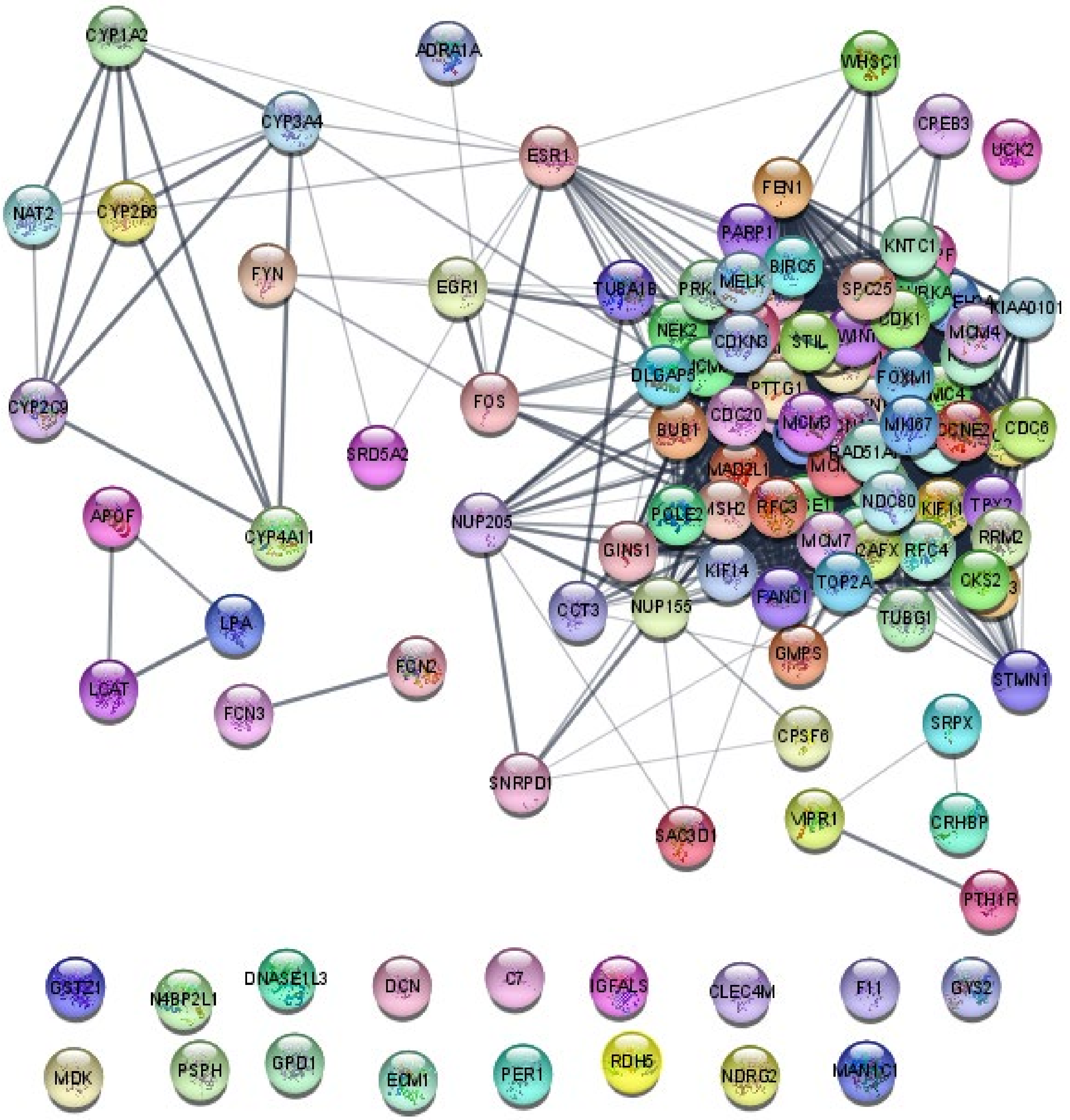

Figure S3: Protein-protein interaction (PPI) network of hub genes that were identified by Cytoscape.

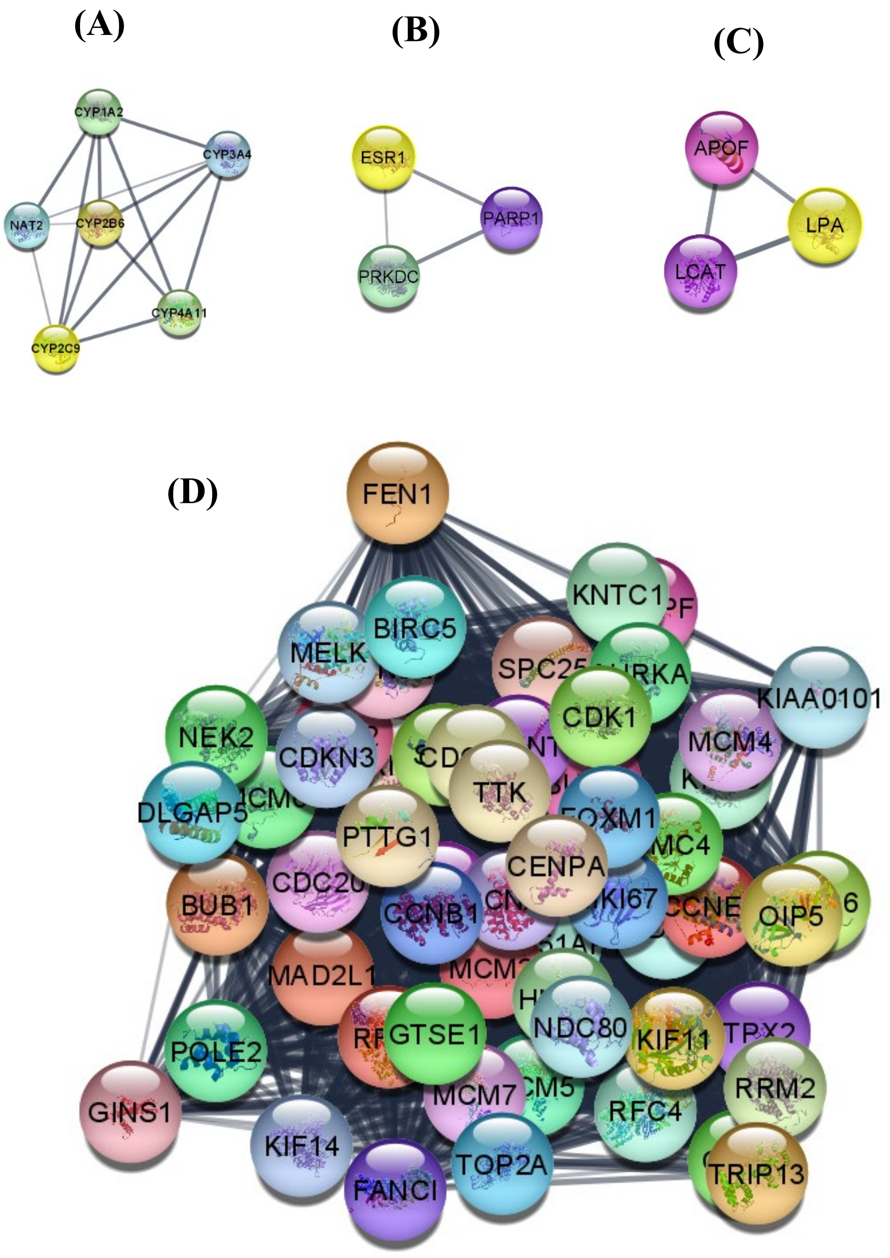

Figure S4: Modules of protein-protein interaction (PPI) network. Four modules (**A-D**) were identified by Cytoscape.

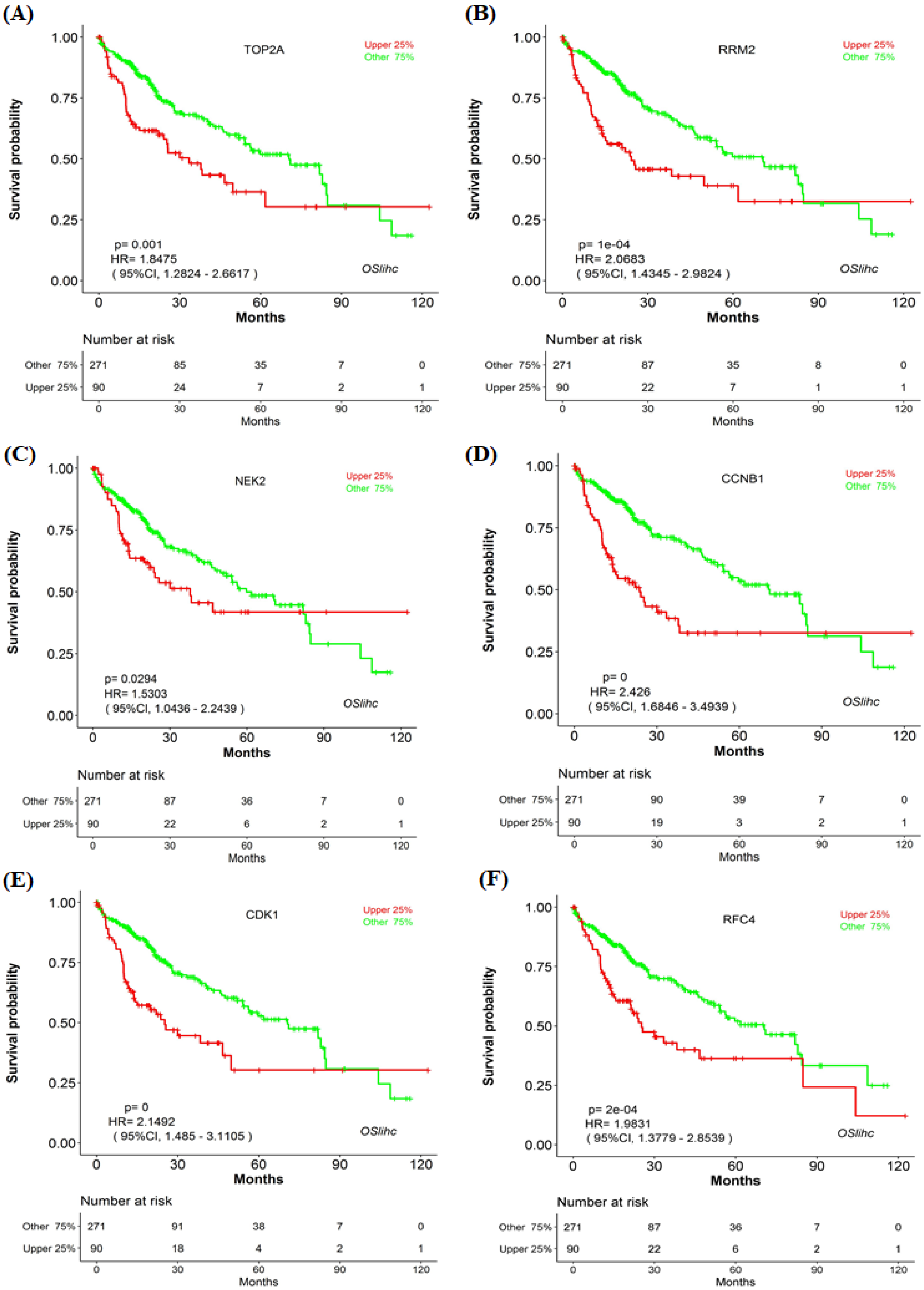

(A)
TOP2A
Upper 25%
Other 75%
Survival probability
1.00
0.75
0.50
0.25
0.00
p= 0.001
HR= 1.8475
(95%CI, 1.2824 - 2.6617)
OSlihc
0 30 60 90 120
Months
Number at risk
Other 75% 271 85 35 7 0
Upper 25% 90 24 7 2 1
0 30 60 90 120
Months
(B)
RRM2
Upper 25%
Other 75%
Survival probability
p= 1e-04
HR= 2.0683
(95%CI, 1.4345 - 2.9824)
OSlihc
Months
Number at risk
Other 75% 271 87 35 8 0
Upper 25% 90 22 7 1 1
Months
(C)
NEK2
Upper 25%
Other 75%
Survival probability
p= 0.0294
HR= 1.5303
(95%CI, 1.0436 - 2.2439)
OSlihc
Months
Number at risk
Other 75% 271 87 36 7 0
Upper 25% 90 22 6 2 1
Months
(D)
CCNB1
Upper 25%
Other 75%
Survival probability
p= 0
HR= 2.426
(95%CI, 1.6846 - 3.4939)
OSlihc
Months
Number at risk
Other 75% 271 90 39 7 0
Upper 25% 90 19 3 2 1
Months
(E)
CDK1
Upper 25%
Other 75%
Survival probability
p= 0
HR= 2.1492
(95%CI, 1.485 - 3.1105)
OSlihc
Months
Number at risk
Other 75% 271 91 38 7 0
Upper 25% 90 18 4 2 1
Months
(F)
RFC4
Upper 25%
Other 75%
Survival probability
p= 2e-04
HR= 1.9831
(95%CI, 1.3779 - 2.8539)
OSlihc
Months
Number at risk
Other 75% 271 87 36 7 0
Upper 25% 90 22 6 2 1
Months

(G)

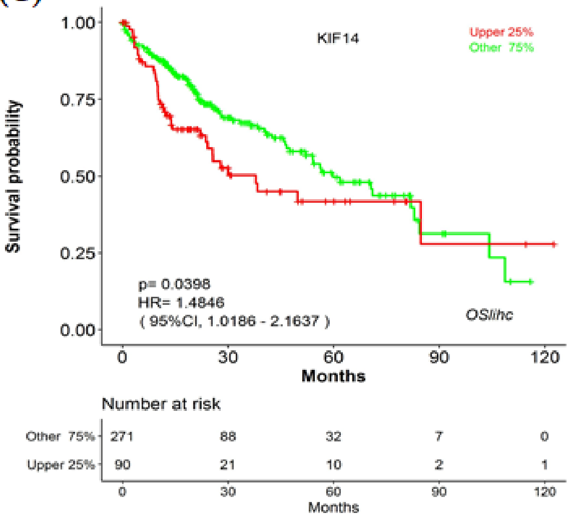

(H)

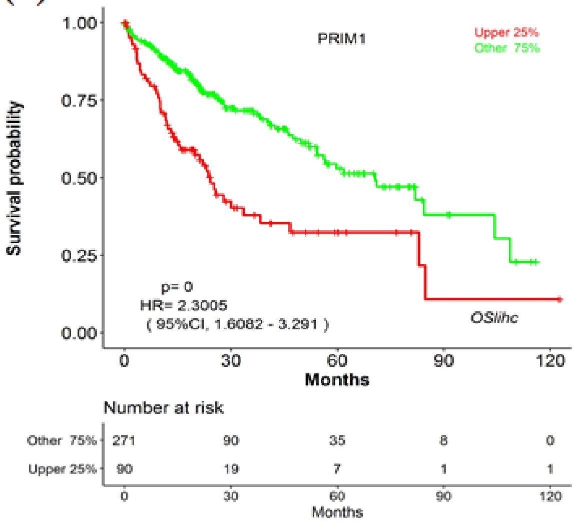

Figure S5: Survival analysis of five hub genes and three other genes of the top ten genes with the highest intramodular connectivity in HCC in OSlihc. The x-axis represents survival time (months) and the y-axis represents survival rate. $p = 0$ indicates $p < 0.0001$. **(A)** *TOP2A*; **(B)** *RRM2*; **(C)** *NEK2*; **(D)** *CCNB1*; **(E)** *CDK1*; **(F)** *RFC4*; **(G)** *KIF14*; **(H)** *PRIM1*.

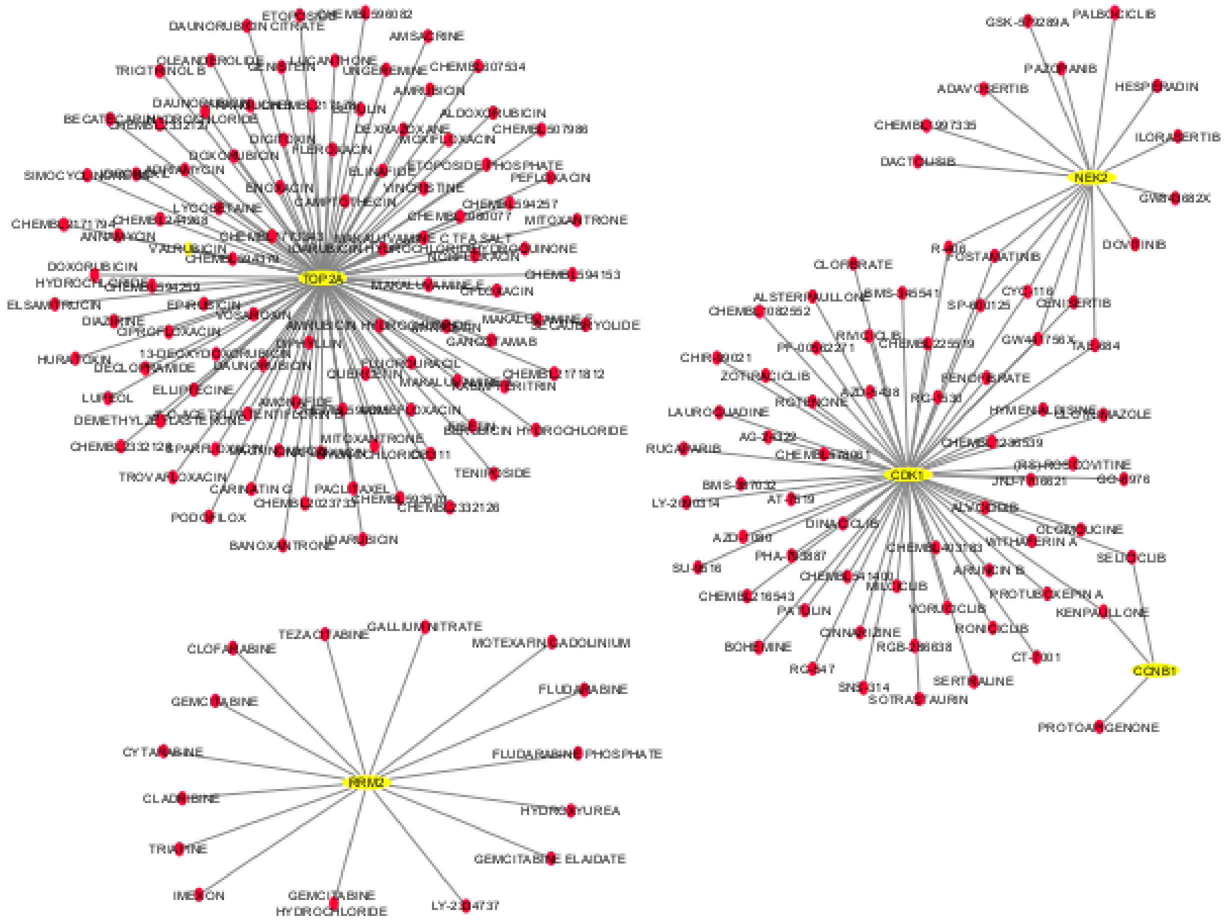

Figure S6: Drug-genes interaction network. Drugs are denoted in red, Drugs-genes interactions are in gray, and genes are in yellow.

Supporting tables:

Table S1: Comparison of the log2(count) values of the top ten Kin genes of HCC patients versus control.

Genes	Control	HCC Patient	*p value*		FC
	**(*n* = 220)**	**(*n* = 225)**	Wilcoxon rank sum t test	Permutation test	
CENPA	1.85±0.09	2.25±0.27	1.17E-58	<0.0001	1.313
DBF4	2.06±0.10	2.43±0.21	5.17E-61	<0.0001	1.293
H2AFX	2.50±0.11	2.87±0.18	3.11E-63	<0.0001	1.291
KIAA1794	1.95±0.1	2.32±0.24	<2E-16	<0.0001	1.294
KIF14	1.90±0.10	2.22±0.22	3.57E-52	<0.0001	1.249
NEK2	1.86±0.10	2.41±0.29	6.33E-67	<0.0001	1.468
PRIM1	2.09±0.12	2.52±0.23	8.58E-57	<0.0001	1.341
RFC4	2.27±0.14	2.80±0.23	7.28E-63	<0.0001	1.443
RRM2	1.94±0.23	2.77±0.32	1.65E-64	<0.0001	1.785
TOP2A	1.94±0.18	2.75±0.35	2.60E-63	<0.0001	1.686

Data were presented as mean ± SD of the log2(count); HCC: Hepatocellular carcinoma, FC: Fold change.

Table S2: Degree of proteins in protein-protein network by Cytoscape.

**Protein**	**Degree**	**Protein**	**Degree**
CDK1	70	MSH2	36
CCNB1	68	DBF4	35
TOP2A	67	STIL	35
CCNA2	65	ESR1	23
MAD2L1	65	TUBG1	23
RFC4	65	NUP155	20
AURKA	64	STMN1	20
BUB1B	64	PARP1	19
CDC20	64	PRKDC	17
CCNB2	63	GMPS	12
KIF11	63	NUP205	12
MCM4	62	TUBA1B	12
BIRC5	61	FOS	11

BUB1	61	CYP3A4	8
CDC6	61	CCT3	7
NDC80	61	WHSC1	7
TPX2	61	CYP1A2	6
CDKN3	60	CYP2B6	6
FEN1	60	CYP2C9	5
MCM2	60	SNRPD1	5
MCM3	60	CYP4A11	4
RRM2	60	EGR1	4
TTK	60	NAT2	4
FANCI	59	CPEB3	3
KIF2C	59	FYN	3
SMC4	59	SAC3D1	3
CENPA	58	APOF	2
DLGAP5	58	CPSF6	2
MCM7	58	LCAT	2
MELK	58	LPA	2
POLE2	58	SRD5A2	2
TRIP13	58	SRPX	2
MCM6	57	VIPR1	2
ZWINT	57	ADRA1A	1
CENPF	56	CRHBP	1
PTTG1	56	FCN2	1
RAD51AP1	56	FCN3	1
KNTC1	55	PTH1R	1
KIAA0101	54	UCK2	1
MKI67	54	C7	0
OIP5	54	CLEC4M	0
MCM5	53	DCN	0
PRIM1	53	DNASE1L3	0

FOXM1	52	ECM1	0
HMMR	52	F11	0
CDC25C	51	GPD1	0
NEK2	51	GSTZ1	0
RFC3	51	GYS2	0
SPC25	50	IGFALS	0
CKS2	49	MAN1C1	0
KIF14	49	MDK	0
CCNE2	48	N4BP2L1	0
CDC7	47	NDRG2	0
EZH2	47	PER1	0
GTSE1	45	PSPH	0
GINS1	40	RDH5	0
RNASEH2A	39		
H2AFX	36		

Table S3: The association of the hub genes and overall survival in the TCGA_LIHC dataset.

Symbol	Univariate Cox regression analysis		
	HR	95% CI	p-value
CCNB1	1.958	(1.37-2.80)	<0.001
CDK1	1.751	(1.23-2.49)	0.002
KIF14	1.627	(1.15-2.31)	0.006
NEK2	2.057	(1.44-2.94)	<0.001
PRIM1	1.549	(1.10-2.19)	0.013
RFC4	1.812	(1.28-2.58)	<0.001
RRM2	1.769	(1.25-2.51)	0.001
TOP2A	1.715	(1.21-2.43)	0.002

HR: Hazard ratio; CI: confident interval; TCGA: The Cancer Genome Atlas; LIHC: Liver Hepatocellular Carcinoma
